# Molecular Profiles of Sensitization to Non-Specific Lipid Transfer Proteins in Lithuania: Single Center Experience

**DOI:** 10.3390/ijms252413535

**Published:** 2024-12-18

**Authors:** Sandra Sakalauskaite, Ligita Pilkyte, Edita Gasiuniene, Brigita Gradauskiene

**Affiliations:** 1 Laboratory of Immunology, Department of Immunology and Allergology, Lithuanian University of Health Sciences, 50161 Kaunas, Lithuania; sandra.sakalauskaite@lsmu.lt (S.S.); edita.gasiuniene@lsmu.lt (E.G.); 2 Department of Immunology and Allergology, Lithuanian University of Health Sciences, 50161 Kaunas, Lithuania; ligita.pilkyte@lsmu.lt

**Keywords:** allergen extracts, anaphylaxis, food allergy, molecular allergy diagnostics, nsLTP, sensitization, systemic reactions

## Abstract

Non-specific Lipid Transfer proteins (nsLTPs) are relevant allergens of several pollens and plant foods. Sensitization to nsLTPs is not typical in our region. Still, it has become an increasingly common cause of IgE-mediated food allergies and food-induced anaphylaxis in Northern Europe in recent decades. No in-depth studies describe the prevalence of sensitization of molecular components to nsLTPs in Lithuania. This study aimed to determine the sensitization profile of atopic patients at the Immunology and Allergy Department of Kauno Klinikos to the components of nsLTPs, using molecular allergen component analysis. Sixty Lithuanian adults with symptoms of allergic rhinitis and/or allergic asthma and/or food allergies were included into the study. Specific immunoglobulin E (IgE) levels were measured using two in vitro techniques: allergen extract and molecular component analysis. Results showed that 25% of subjects were sensitized to nsLTP-containing allergen sources, mostly to Zea m 14, Mal d 3, Vit v 1, and Art v 3. The median amount of total IgE was higher in nsLTP-sensitized patients than in nsLTP-nonsensitized patients. Based on Cohen’s Kappa and McNemar tests, the results of allergen extract and component analysis tests do not always agree, especially when we determine the sensitization to allergen sources containing nsLTPs. Molecular allergen component analysis could be the first choice in determining detailed sensitization to nsLTPs in patients who experienced anaphylaxis of unknown origin.

## 1. Introduction

Allergy is Europe’s most common chronic disease, affecting a significant portion of the population [1]. According to the World Allergy Organization (WAO), the prevalence of allergic diseases across different countries varies between 10% and 40%, depending on various environmental and genetic factors. Allergic diseases can range from mild conditions such as rhinitis to severe cases such as certain types of asthma, food allergies and anaphylaxis [2,3]. Furthermore, allergic conditions often co-occur within individuals, making an integrated approach essential for effective diagnosis and treatment [4].

In vivo and in vitro tests are used for accurate allergy diagnosis and detailed information about exposure to the supposed allergen of IgE-mediated diseases. The most reliable and cost-effective tool in vivo is skin tests, especially a Skin Prick Test (SPT) [5]. For this test, based on the specific clinical history, allergen exposure pattern, distribution of allergenic sources in the local environment, and living conditions, a mixture of allergen extracts is chosen for diagnosing suspectable allergens [6]. Traditional immunological diagnosis in vitro tools using allergen extracts are based on determining specific IgE (sIgE) antibodies for extracts of native allergens. Allergen extracts derived from natural allergen sources are typically heterogeneous, often containing various non-allergenic molecules [7]. In recent decades, there has been significant progress in molecular allergy diagnostic tools in vitro allowing the detection of sIgE in the blood against single allergen components from a specific allergen source [8]. Single allergens rather than extracts offer several advantages, including improved assay performance and enhanced interpretative capabilities, such as risk assessment and differentiation between true sensitization and cross-reactivity, especially in polysensitized patients, prediction of reaction severity—such as the risk of severe anaphylaxis—and estimation of the likely efficacy of allergen immunotherapy [9,10].

Usually, sensitization to specific pollen allergens is characterized by cross-reactivity with food allergens, and it is currently known as Pollen-Food Allergy Syndrome (PFAS) [11]. Previously, localized oral manifestation of unstable allergens observed after consumption of certain fruits and vegetables was understood in individuals with pollinosis as Oral Allergy Symptoms (OASs) [12]. OASs comprise only symptoms localized to the oral cavity. At the same time, PFAS more broadly includes food allergies and causes symptoms that can vary from itching, numbness, and mucosal edema of the lips or mouth to respiratory (rhinitis) and gastrointestinal symptoms and, rarely, systemic manifestations (anaphylaxis) [13,14]. Non-specific Lipid Transfer Proteins (nsLTPs) are relevant allergens found in several pollens and plant foods belonging to the widespread Rosaceae family and other distantly related species. In the last 20 years, sensitization to nsLTPs and consequent reactions to plant foods have become an increasing concern [15]. These allergens can be found in fruits, vegetables, nuts, seeds, legumes, and cereals, and research has demonstrated that food nsLTPs can cause sensitization via oral, respiratory, and skin exposure [16]. Symptoms can vary significantly, ranging from no reaction to PFAS, contact urticaria, asthma, hives/angioedema, and food-dependent exercise-induced anaphylaxis [17,18]. Although nsLTPs are the most common cause of primary food allergies among adults in Mediterranean countries and are also the primary trigger for food-induced anaphylaxis in these regions, sensitization to nsLTPs is emerging in other European countries [9]. Sensitization rates to nsLTPs in selected children and adults with pollen and/or plant food allergies exceeded 20% in Belgium [19]. Not all proteins in this family develop strong cross-reactions and can elicit systemic reactions in nsLTP-sensitized patients, so an accurate diagnosis in determining the sensitization to nsLTPs is crucial.

Sensitization to nsLTPs has been extensively studied in some countries, but there is limited data on sensitization profiles in Northern and Eastern Europe, especially in the Baltic region. No in-depth studies describe the prevalence of sensitization of molecular components to nsLTPs in Lithuania. Furthermore, there is evidence that in areas with high exposure to pollen, particularly regarding mugwort (as in Lithuania), pollen-derived nsLTPs can act as a primary sensitizer to trigger secondary food allergies [20]. Thus, this study aimed to determine the sensitization profile of atopic patients at the Immunology and Allergy Department of Kauno Klinikos regarding the components of nsLTPs using molecular allergen component analysis.

## 2. Results

### 2.1. The Main Allergen Sources of the Study Group

The description of this prospective study group is presented in Table 1. nsLTP-sensitized patients mostly had an AR diagnosis (75.00%): 16.67% were AD + AR and only 8.33% were AA + AR.

**Figure 1:**
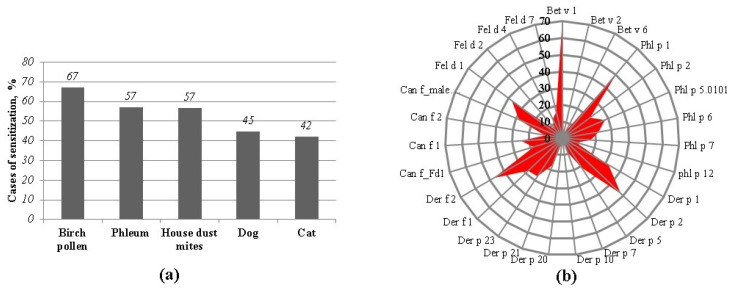
The prevalence of sensitization of the whole allergen source (**a**) and their separate molecular components (**b**) in studied group. Cases of sensitization indicated in both parts—%.

The most prevalent allergen sources were determined using a molecular allergy diagnostic system. The subjects were mostly sensitized to Birch pollen (67%) (see Figure 1). All of them were sensitized to the component Bet v 1, and only 5.2% and 3.4% were sensitized to the minor allergens Bet v 6 and Bet v 2, respectively. Almost 60.0% of patients were sensitized to Phleum. Phl p 1 (50.0%) and Phl p 5.0101 (27.8%) were the main determined components. The most common allergen components to which subjects were sensitized to regarding house dust mites were Der p 2 (48.2%), Der f 2 (47.4%), and Der p 23 (27.1%). Sensitization to dog allergens was higher than to the cat allergens: 45.0% and 42.0%, respectively. Despite this, patients were sensitized to different dog allergen components, while the most prevalent component of the cat allergens was Fel d 1 (37.5%).

### 2.2. Sensitization to nsLTP-Containing Allergen Sources

**Figure 2:**
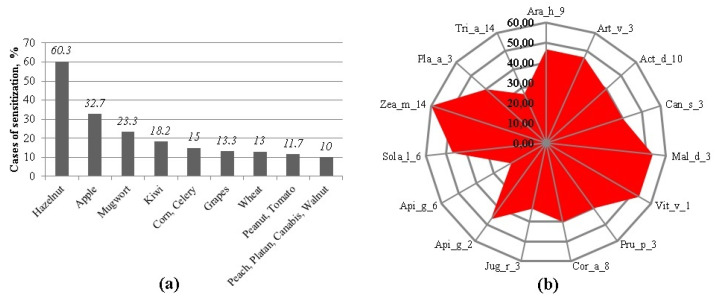
The prevalence of sensitization to nsLTP-containing allergen sources (**a**) and to different nsLTPs (**b**). Data presented as percentage of the whole group (**a**) and percentage of nsLTP-sensitized subjects (**b**).

The main nsLTP-containing allergen sources determined were hazelnut (60.0%), apple (32.7%), and mugwort (23.3%). Sensitization to other nsLTP-containing allergen sources was less than 20.0% (see Figure 2). Fifteen percent of the studied subjects were sensitized to corn; its component Zea m 14 (60.00%) was the most prevalent in nsLTP-sensitized patients. Two other components, to which 53.33% of patients were sensitized, were Mal d 3 (from apple) and Vit v 1 (from grapes). About 47.00% of nsLTP-sensitized patients were sensitized to Ara h 9 (from hazelnut), Art v 3 (from mugwort), Sol l 6 (from tomato), and Api g 2 (from celery). On the other hand, only 20.00% of subjects were sensitized to the component of celery—Api g 6. Sensitization to kiwi and peach components Act d 10 and Pru p 3, respectively, reached 40%. Thirty and fewer percent of subjects were sensitized to Jug r 3 (from walnut) and Tri a 14 (from wheat) components.

**Figure 3:**
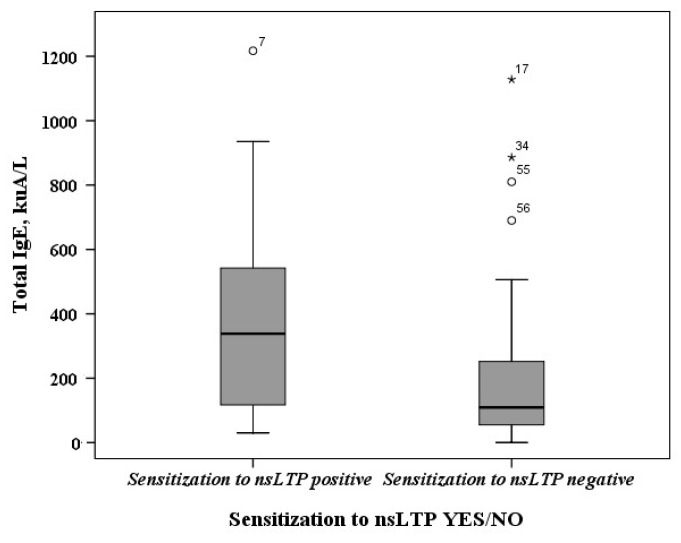
The amount of total IgE in nsLTP-sensitized subjects or without sensitization to nsLTPs (minimum data value, lower quartile value, median value, upper quartile value, maximum data value, outliers).

It was determined that the median of the total IgE amount was higher in subjects who were sensitized to one or more nsLTPs components than in subjects without sensitization to nsLTP-containing sources (see Figure 3). The median of the total IgE was 338 kuA/L in nsLTP-sensitized patients and 109 kUA/L in the patients without sensitization to nsLTPs.

### 2.3. The Determination of Sensitization Profiles to nsLTPs

**Figure 4:**
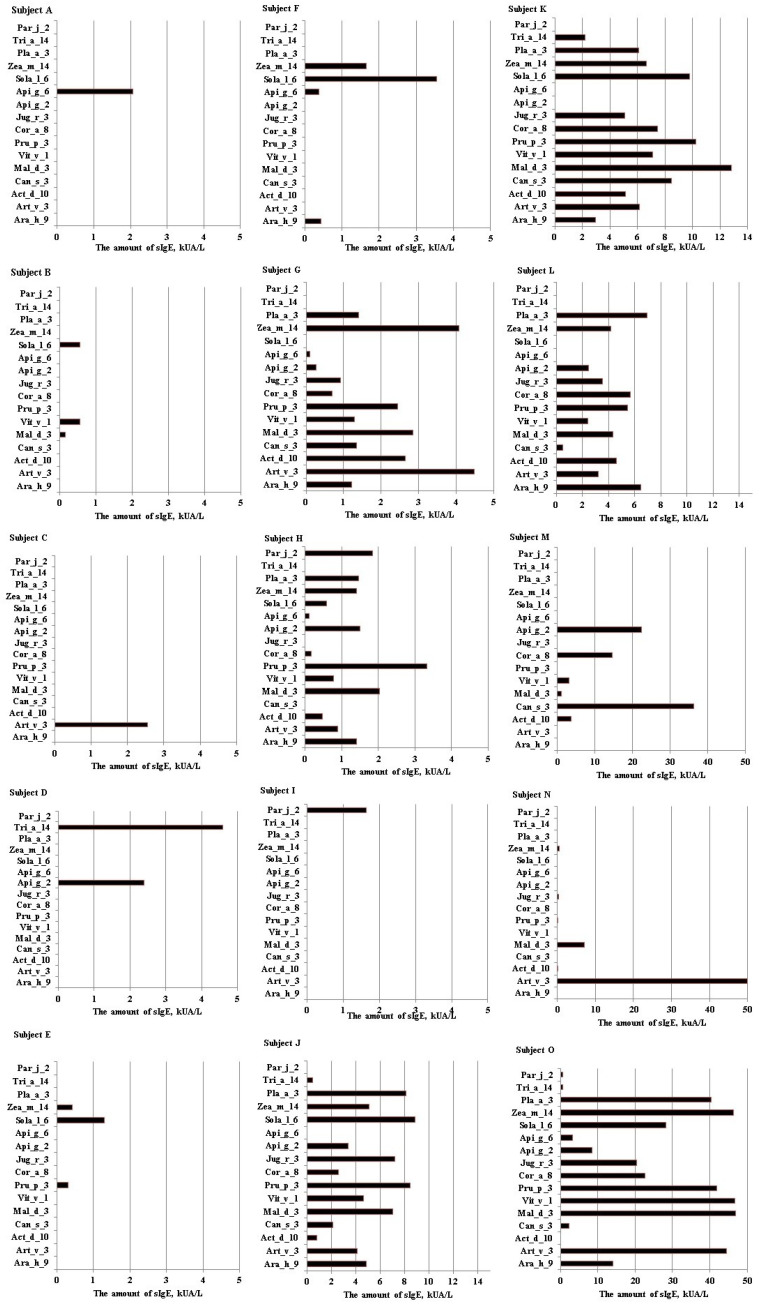
The sensitization profiles to nsLTP components. Different patients indicated as (**A**–**O**). The concentration of sIgE up to 5 kUA/L (**A**–**I**), up to 15 kUA/L (**J**–**L**), and up to 50 (**M**–**O**).

The analysis of the sIgE concentration showed that subjects were sensitized to different numbers of nsLTP components (from 1 to 14) (see Figure 4). The concentration of sIgE varied from 0.6 kUA/L to 50 kUA/L. The highest sIgE values were against Mal d 3 (median 4.33), Art v 3 (median 4.13), and Zea m 14 (median 4.07). Furthermore, the amount of sIgE was not associated with the number of components to which subjects were sensitized (*p* = 0.445). In addition, it is clear from the medical records that all these nsLTP-sensitized subjects had experienced anaphylaxis of unknown origin.

### 2.4. The Comparison of Allergen Extracts and Component Analysis Results in Determining Sensitization to nsLTPs

The agreement of the sensitization results to allergen sources containing nsLTP or PR-10 components obtained via two different methods, allergen extracts and component analysis, was assessed based on Cohen’s Kappa coefficient (κ) and the McNemar test results (See Table 2). The obtained results between the allergen extract and component analysis methods did not differ largely. They were in moderate agreement when evaluating sensitization to Cor a 1.0103 (κ = 0.532), Cor a 1.0401 (κ = 0.532) (belonging to the PR-10 family), Sol l 6 (κ = 0.612), and Ara h 9 (κ = 0.542) (belonging to the nsLTP family) components. Fair agreement and, based on the *p*-values of the McNemar test, no statistically significant differences were determined between the results obtained via the allergen extract and component analysis methods evaluating sensitization to Mal d 1, which belongs to PR-10 proteins (κ = 0.398). Additionally, fair agreement (κ = 0.341) but with statistically significant differences (the *p*-value of the McNemar test = 0.001) was found in sensitization determination to Mal d 3 and Art v 3 (κ = 0.259) components (belonging to nsLTPs) between the allergen extract method and the component analysis method. No agreement was found in the sensitization to Ara h 8 determination between the allergen extract and component analysis test results (κ = −0.101). The agreement between the allergen extract and component analysis methods when determining sensitization to celery components (Api g 2, Api g 6, and Api g 1) was weak and coincidental (*p* > 0.05). However, substantial agreement was found between sensitization to celery determined via the allergen extract test and sensitization to nsLTP components determined via the component analysis test (κ = 0.612 and the *p*-value of the McNemar test = 1).

## 3. Discussion

The aim of the study was to determine the sensitization profile to nsLTPs of atopic patients at the Immunology and Allergy Department of Kauno Klinikos using molecular allergen component analysis. This is the first study describing the molecular sensitization profiles to nsLTP family proteins in Lithuania and one of the few studies conducted in Northern Europe. Our results coincide with other studies [14,16], which showed that clinically relevant sensitization occurs in Northern Europe, with nsLTP sensitization profiles broadly similar to Mediterranean countries.

Our results revealed the allergen sensitization profiles of the subjects studied with atopic diseases. The most prevalent allergen sources were pollen (Birch pollen and Phelum) and its components Bet v 1 and Phl p 1, respectively. About 48% subjects were sensitized to house dust mite allergens (components: Der p 2 and Der f 2). A quarter of subjects with atopic diseases (25%) were sensitized to nsLTP-containing allergen sources. Zea m 14 (60%) and Mal d 3 (from apple) and Vit v 1 (from grapes) (53.33%) were the most prevalent. About 47% of patients were sensitized to Ara h 9 (from hazelnut), Art v 3 (from mugwort), Sol l 6 (from tomato), and Api g 2 (from celery). Based on anamnesis of the medical history, all nsLTP-sensitized patients had experienced systemic reactions of unknown origin. We found that the median of the total IgE was higher in nsLTP-sensitized patients than in patients without sensitization to nsLTPs. In a study conducted by Polish researchers, higher total IgE values were also observed in subjects sensitized to the components of nsLTPs than in those not sensitized to nsLTPs [14]. Additionally, we found that sensitization to celery determined via the allergen extract method and sensitization to nsLTPs determined via the component analysis method were in substantial agreement. This suggests, that if sensitization to celery extract is determined, there is a high probability that the subject is sensitized to nsLTP components. Therefore, if we determine the sensitization to celery using allergen extracts, this does not mean that the patient is truly sensitized to celery. Still, it can indicate that the patient is sensitized to nsLTP family proteins. Such results only confirm that conventional in vitro allergy testing, which relies on allergen extracts, can identify the general source of sensitizing allergens but lacks the precision to detect cross-reactivities or predict the potential severity of a reaction after allergen exposure. This limitation is particularly critical for individuals with food allergies [7,21].

Usually, manufacturers do not indicate the quality of the components in the extract, so there may be differences in sensitization profiles. Furthermore, standardizing the allergen extracts from fruits and vegetables is difficult because they may contain varying amounts of nsLTPs due to the differences in species and growing, ripening, or storage conditions [21,22]. Meanwhile, molecular allergy diagnostics are based on the detection of sIgE in multiple individual allergen components [23]. This is reflected in our results. The Cohen’s Kappa and McNemar test results of our research revealed that the results of two different allergy diagnostics tests (allergen extracts and component analysis) do not always agree, especially when we determine the sensitization to allergen sources containing nsLTPs. The determination of sensitization to apple extract, the Mal d 3 component or mugwort extract, the Art v 3 component and celery extract, and the Api g 6 component, using allergen extracts or component analysis tests, had statistically significant differences. On the other hand, in the sensitization determination regarding allergens belonging to the PR-10 family, the results from allergen extract and component analysis tests were in moderate or fair agreement (Cor a 1.0103, Cor a 1.10401, and Mal d 1). This suggests that if we determine the sensitization to the allergen extract, which consists of a mixture of PR-10 and nsLTP family proteins, we will not be able to distinguish whether patients are sensitized to nsLTPs or PR-10 family proteins, and it is not clear how severe the reactions of a sensitized patient might be. Furthermore, studies showed that molecular diagnostics helped to reduce the number of anaphylaxis cases with an unknown cause by identifying hidden allergens that were previously undetectable [24]. Other researchers have also noted the advantage of component analysis rather than extract tests in patients with pollen and food sensitization [11,25].

In Southern Europe, sensitization to nsLTPs is primarily associated with peach (Pru p 3). sIgE responses to nsLTPs are rarely observed without the presence of Pru p 3, and sIgE levels against it are typically the highest among all nsLTPs. Due to its significant clinical relevance, this leads to a consensus that peach component Pru p 3 is the key marker (as a primary sensitizer) for LTP-driven allergies in the Mediterranean region [16,20,26,27]. Meanwhile, the main sensitizer belonging to the nsLTP family in Northern Europe is still unknown. Some studies show that mugwort is an especially common allergen source of nsLTPs in Northern Europe. Furthermore, greater attention should be paid to cannabis allergens, which are showing an increasing incidence of sensitization in Central Europe [14,28]. However, Zea m 14 (60%), Mal d 3, and Vit v 1 (53.3%) were the most prevalent nsLTP family components in our study. The highest sIgE levels were determined against Mal d 3. sIgE against Art v 3 from mugwort was determined in 47% of nsLTP-sensitized patients, and the sIgE values were slightly lower than against Mal d 3. Components Sol l 6 and Api g 2 were prevalent at the same level as Art v 3. Sensitization to Act d 10, Pru p 3, and Can s 3 was determined in 40% of nsLTP-sensitized patients. A very similar nsLTP-sensitized allergen profile was described in an unusual case report from Germany [29]. Ara h 9 from peanuts is clinically relevant to Northern European populations and is associated with bronchospasm [30]. According to the study results in Lithuania, sensitization to the Ara h 9 component is very rare in children [31]. The results of our study carried out with adults showed that 47% of nsLTP-sensitized patients were sensitized to Ara h 9. It can therefore be assumed that the sensitization to Ara h 9 develops with aging. Such age-dependent sensitization patterns were observed in Calamelli et al.’s study [32].

One possible limitation of our study is that the findings are based on the results from a single center experience and a small sample size. Nevertheless, this is the first study that analyzes Lithuanian patients’ sensitization profiles to nsLTP family components that may cause systemic reactions. Additionally, knowing that sensitization to nsLTPs components is not typical in our region, we found significant results that showed a wide range of nsLTP components to which patients are sensitized. Such results presuppose allergologists select an appropriate molecular allergy in vitro test for patients with a history of anaphylaxis of uncertain origin.

## 4. Materials and Methods

The prospective study was carried out to evaluate the prevalence of sensitization to nsLTPs in atopic patients from the Department of Immunology and Allergology in the Hospital of Lithuanian University for Health Sciences Kauno Klinikos, which is the largest hospital in Lithuania. Data were collected from February 2022 to January 2024. The study was approved by the Bioethical Committee on 10 February 2022 (No. BE-2-3). Sixty adults with allergic rhinitis with/without allergic asthma and who have had suspected food-related allergic reactions (oral allergy syndrome/pollen food allergy syndrome or anaphylaxis) (diagnosed according to the GINA [33], ARIA [34], and GRADE [35] recommendations) were included in the study. Anamnesis about allergy-related conditions was collected. The concentration of allergen-specific immunoglobulin E (sIgE) in serum was determined via two in vitro diagnostic systems: using allergen extracts with multiplex immunoblot assay an atopy pallet of Euroline (EUROIMMUN, Lubak, Germany) and component analysis using an Elisa-based multiplex allergy test—Allergy Explorer, Alex^2^ (Macro Array Diagnostics GmbH, Vienna, Austria). The Alex^2^ multiplex tool enables the detection of sIgE in allergen extracts and multiple individual allergen components. It allows clinicians to distinguish clinically relevant sensitizations and cross-reactivity. The ability to determine the sensitization to nsLTPs via these two methods was compared binarily (positive vs. negative). According to the manufacturer’s instructions, the sensitization was defined by detecting a sIgE level greater than 0.35 kUA/L using an allergen extract test, and 0.3 kUA/L using a molecular allergen component analysis test.

### Statistical Analysis

IBM SPSS Statistics for Windows (version 29.0.2.0; SPSS Inc., Chicago, IL, USA) was used. Categorical data were reported as percentages indicating the proportion of positive results. According to data distribution, continuous variables have been described as mean with standard deviation or median. The results were considered statistically significant when *p* < 0.05. Paired comparisons within two allergy diagnostic tests (using allergen extracts or components) were made using Cohen’s Kappa (κ) and the McNemar test to determine the overlap/differences between methods and their significance. Non-random overlap between allergen extract and molecular analysis tests was considered when the *p*-value of Cohen’s Kappa (κ) < 0.05. Statistically significant differences were considered when the *p*-value of McNemar’s test < 0.05.

## 5. Conclusions

A quarter of atopic subjects studied in our center were sensitized to nsLTPs containing allergen sources, of which the most prevalent were Zea m 14, Mal d 3, Vit v 1, and Art v 3. An accurate diagnosis of sensitization to the components of nsLTPs is clinically relevant in patients who experience systemic reactions (anaphylaxis) of unknown origin. Therefore, molecular allergen component analysis could be the first choice in determining detailed sensitization to nsLTPs in these patients.

## Figures and Tables

**Table 1:** Description of the study group.

Gender	The Part of Population (n = 60), %
Man	45.00
Woman	55.00
Age mean (min–max), m	33.58 (18–60)
Diagnosis	
AD + AR	12.20
AR	58.50
AA + AR	29.30

**Table 2:** Comparison of the sensitization to nsLTPs and PR-10 family protein results obtained using allergen extract and component analysis techniques.

			Molecular Component				
Protein Family		nsLTP			PR-10		
Allergen Source		κ	*p*-Value	*p*-Value of McNemar’s Test	κ	*p*-Value	*p*-Value of McNemar’s Test
Allergen extracts	Hazelnut	Cor a 8			Cor a 1.0103		
		−0.118	0.581	0.508	0.532	0.024	1
					Cor a 1.0401		
					0.538	0.019	0.625
	Apple	Mal d 3			Mal d 1		
		0.341	0.001	0.001	0.389	0.004	1
	Peanut	Ara h 9			Ara h 8		
		0.542	0.001	1	−0.101	0.309	0.021
	Celery	Api g 2			Api g 1		
		0.379	0.075	0.375	0.286	0.180	0.375
		Api g 6					
		0.174	0.179	0.031			
	Kiwi	Act d 10			-		
		0.301	0.201	1			
	Tomato	Sol l 6			-		
		0.612	0.004	0.5			
	Mugwort	Art v 3			-		
		0.259	0.004	0.001			

## Data Availability

Data is contained within the article.

## References

[1] Seastedt H., Nadeau K. (2023). Factors by Which Global Warming Worsens Allergic Disease. Ann. Allergy Asthma Immunol..

[2] Bonini S. (2000). News From the European Academy of Allergology and Clinical Immunology. Allergy Clin. Immunol. Int. J. World Allergy Organ..

[3] Pawankar R. (2014). Allergic Diseases and Asthma: A Global Public Health Concern and a Call to Action. World Allergy Organ. J..

[4] Skypala I.J., De Jong N.W., Angier E., Gardner J., Kull I., Ryan D., Venter C., Vlieg-Boerstra B.J., Grimshaw K. (2018). Promoting and Achieving Excellence in the Delivery of Integrated Allergy Care: The European Academy of Allergy & Clinical Immunology Competencies for Allied Health Professionals Working in Allergy. Clin. Transl. Allergy.

[5] Van Der Valk J.P.M., Gerth Van Wijk R., Hoorn E., Groenendijk L., Groenendijk I.M., De Jong N.W. (2016). Measurement and Interpretation of Skin Prick Test Results. Clin. Transl. Allergy.

[6] Ansotegui I.J., Melioli G., Canonica G.W., Caraballo L., Villa E., Ebisawa M., Passalacqua G., Savi E., Ebo D., Gómez R.M. (2020). IgE Allergy Diagnostics and Other Relevant Tests in Allergy, a World Allergy Organization Position Paper. World Allergy Organ. J..

[7] Lis K., Bartuzi Z. (2023). Selected Technical Aspects of Molecular Allergy Diagnostics. Curr. Issues Mol. Biol..

[8] Luengo O., Labrador-Horrillo M. (2022). Molecular Allergy Diagnosis in Clinical Practice: Frequently Asked Questions. J. Investig. Allergol. Clin. Immunol..

[9] Werfel T., Asero R., Ballmer-Weber B.K., Beyer K., Enrique E., Knulst A.C., Mari A., Muraro A., Ollert M., Poulsen L.K. (2015). Position Paper of the EAACI: Food Allergy Due to Immunological Cross-Reactions with Common Inhalant Allergens. Allergy.

[10] Passalacqua G., Melioli G., Bonifazi F., Bonini S., Maggi E., Senna G., Triggiani M., Nettis E., Rossi R.E., Vacca A. (2013). The Additional Values of Microarray Allergen Assay in the Management of Polysensitized Patients with Respiratory Allergy. Allergy.

[11] Kato Y., Morikawa T., Fujieda S. (2024). Comprehensive Review of Pollen-Food Allergy Syndrome: Pathogenesis, Epidemiology, and Treatment Approaches. Allergol. Int..

[12] Carlson G., Coop C. (2019). Pollen Food Allergy Syndrome (PFAS): A Review of Current Available Literature. Ann. Allergy Asthma Immunol..

[13] Kondo Y., Urisu A. (2009). Oral Allergy Syndrome. Allergol. Int..

[14] Wąsik J., Likońska A., Kurowski M. (2024). IgE-Mediated Allergy and Asymptomatic Sensitization to Cannabis Allergens—Review of Current Knowledge and Presentation of Six Cases. Medicina.

[15] Lin C.H., Li L., Lyu P.C., Chang J.Y. (2004). Distinct Unfolding and Refolding Pathways of Lipid Transfer Proteins LTP1 and LTP2. Protein J..

[16] Skypala I.J., Asero R., Barber D., Cecchi L., Diaz Perales A., Hoffmann-Sommergruber K., Pastorello E.A., Swoboda I., Bartra J., Ebo D.G. (2021). Non-specific Lipid-transfer Proteins: Allergen Structure and Function, Cross-reactivity, Sensitization, and Epidemiology. Clin. Transl. Allergy.

[17] Romano A., Scala E., Rumi G., Gaeta F., Caruso C., Alonzi C., Maggioletti M., Ferrara R., Palazzo P., Palmieri V. (2012). Lipid Transfer Proteins: The Most Frequent Sensitizer in Italian Subjects with Food-Dependent Exercise-Induced Anaphylaxis. Clin. Exp. Allergy.

[18] Asero R., Pravettoni V., Villalta D., Cecchi L., Scala E. (2024). IgE-Mediated Reactivity to Non-Specific Lipid Transfer Protein (NsLTP): Clinical Implications and Management—A Consensus Document of the Association of Italian Territorial and Hospital Allergists and Immunologists (AAIITO). Eur. Ann. Allergy Clin. Immunol..

[19] Faber M.A., Van Gasse A.L., Decuyper I.I., Uyttebroek A., Sabato V., Hagendorens M.M., Bridts C.H., De Clerck L.S., Fernandez-Rivas M., Pascal M. (2017). IgE-Reactivity Profiles to Nonspecific Lipid Transfer Proteins in a Northwestern European Country. J. Allergy Clin. Immunol..

[20] Scheurer S., van Ree R., Vieths S. (2021). The Role of Lipid Transfer Proteins as Food and Pollen Allergens Outside the Mediterranean Area. Curr. Allergy Asthma Rep..

[21] Ciardiello M.A., Giangrieco I., Tuppo L., Tamburrini M., Buccheri M., Palazzo P., Bernardi M.L., Ferrara R., Mari A. (2009). Influence of the Natural Ripening Stage, Cold Storage, and Ethylene Treatment on the Protein and Ige-Binding Profiles of Green and Gold Kiwi Fruit Extracts. J. Agric. Food Chem..

[22] Skypala I.J., Bartra J., Ebo D.G., Antje Faber M., Fernández-Rivas M., Gomez F., Luengo O., Till S.J., Asero R., Barber D. (2021). The Diagnosis and Management of Allergic Reactions in Patients Sensitized to Non-Specific Lipid Transfer Proteins. Allergy.

[23] Sonneveld L.J.H., Emons J.A.M., Arends N.J.T., Landzaat L.J., Veenbergen S., Schreurs M.W.J. (2022). ALEX versus ISAC Multiplex Array in Analyzing Food Allergy in Atopic Children. Clin. Mol. Allergy.

[24] Poziomkowska-Gęsicka I. (2022). Idiopathic Anaphylaxis? Analysis of Data from the Anaphylaxis Registry for West Pomerania Province, Poland. Int. J. Environ. Res. Public Health.

[25] Quan P.L., Sabaté-Brescó M., D’amelio C.M., Pascal M., García B.E., Gastaminza G., Blanca-López N., Alvarado M.I., Fernández J., Moya C. (2022). Validation of a Commercial Allergen Microarray Platform for Specific Immunoglobulin E Detection of Respiratory and Plant Food Allergens. Ann. Allergy Asthma Immunol..

[26] Scheurer S., Schülke S. (2018). Interaction of Non-Specific Lipid-Transfer Proteins With Plant-Derived Lipids and Its Impact on Allergic Sensitization. Front. Immunol..

[27] Asero R., Pravettoni V., Scala E., Villalta D. (2022). Lipid Transfer Protein Allergy: A Review of Current Con-468troversies. Clin. Exp. Allergy.

[28] Movérare R., Larsson H., Carlsson R., Holmquist I. (2011). Mugwort-Sensitized Individuals from North Europe, South Europe and North America Show Different IgE Reactivity Patterns. Int. Arch. Allergy Immunol..

[29] Albert E., Walsemann T., Behrends J., Jappe U. (2023). Lipid Transfer Protein Syndrome in a Northern European Patient: An Unusual Case Report. Front. Med..

[30] Arkwright P.D., Summers C.W., Riley B.J., Alsediq N., Pumphrey R.S.H. (2013). IgE Sensitization to the Nonspecific Lipid-Transfer Protein Ara h 9 and Peanut-Associated Bronchospasm. BioMed Res. Int..

[31] Adomaite I., Gorbikova E., Miskinyte M., Eidukaite A., Miskiniene A., Rudzeviciene O. (2023). Molecular Peanut Sensitization Patterns in Lithuanian Children with Suspected Allergic Symptoms. Adv. Dermatol. Allergol..

[32] Calamelli E., Caffarelli C., Ricci G. (2013). Peanut Sensitization Profiles in Italian Children and Adolescents with Specific IgE to Peanuts. BioMed Res. Int..

[33] Mauer Y., Do R.M.T. (2020). Managing Adult Asthma: The 2019 GINA Guidelines. Clevel. Clin. J. Med..

[34] Bousquet J., Schünemann H.J., Togias A., Bachert C., Erhola M., Hellings P.W., Klimek L., Pfaar O., Wallace D., Ansotegui I. (2020). Next-Generation Allergic Rhinitis and Its Impact on Asthma (ARIA) Guidelines for Allergic Rhinitis Based on Grading of Recommendations Assessment, Development and Evaluation (GRADE) and Real-World Evidence. J. Allergy Clin. Immunol..

[35] Santos A.F., Riggioni C., Agache I., Akdis C.A., Akdis M., Alvarez-Perea A., Alvaro-Lozano M., Ballmer-Weber B., Barni S., Beyer K. (2023). EAACI Guidelines on the Diagnosis of IgE-Mediated Food Allergy. Allergy.

